# Fingerprint Finder: Identifying Genomic Fingerprint Sites in Cotton Cohorts for Genetic Analysis and Breeding Advancement

**DOI:** 10.3390/genes15030378

**Published:** 2024-03-19

**Authors:** Shang Liu, Hailiang Cheng, Youping Zhang, Man He, Dongyun Zuo, Qiaolian Wang, Limin Lv, Zhongxv Lin, Guoli Song

**Affiliations:** 1 National Key Laboratory of Cotton Bio-breeding and Integrated Utilization, Institute of Cotton Research of Chinese Academy of Agricultural Sciences, Anyang 455000, China; liushang19941115@outlook.com (S.L.); zyp547550790@163.com (Y.Z.); 17637627733@163.com (M.H.); zdy041@163.com (D.Z.); wangql1232021@163.com (Q.W.); llm0372@126.com (L.L.); 2 National Key Laboratory of Crop Genetic Improvement, College of Plant Science and Technology, Huazhong Agricultural University, Wuhan 430070, China; linzhongxu@mail.hzau.edu.cn; 3 Zhengzhou Research Base, Zhengzhou University, Zhengzhou 450001, China

**Keywords:** fingerprint, term frequency–inverse document frequency, cotton genomics, pedigree, geographical bias

## Abstract

Genomic data in Gossypium provide numerous data resources for the cotton genomics community. However, to fill the gap between genomic analysis and breeding field work, detecting the featured genomic items of a subset cohort is essential for geneticists. We developed FPFinder v1.0 software to identify a subset of the cohort’s fingerprint genomic sites. The FPFinder was developed based on the term frequency–inverse document frequency algorithm. With the short-read sequencing of an elite cotton pedigree, we identified 453 pedigree fingerprint genomic sites and found that these pedigree-featured sites had a role in cotton development. In addition, we applied FPFinder to evaluate the geographical bias of fiber-length-related genomic sites from a modern cotton cohort consisting of 410 accessions. Enriching elite sites in cultivars from the Yangtze River region resulted in the longer fiber length of Yangze River-sourced accessions. Apart from characterizing functional sites, we also identified 12,536 region-specific genomic sites. Combining the transcriptome data of multiple tissues and samples under various abiotic stresses, we found that several region-specific sites contributed to environmental adaptation. In this research, FPFinder revealed the role of the cotton pedigree fingerprint and region-specific sites in cotton development and environmental adaptation, respectively. The FPFinder can be applied broadly in other crops and contribute to genetic breeding in the future.

## 1. Introduction

Cotton is an important economic crop and provides the world with the most natural fiber. Thus, improving the cotton fiber’s yield and quality is the main target of cotton breeders [1]. With the development of cotton genomics, a large amount of genome resequencing data was generated to perform genome-wide association analysis (GWAS) [2,3,4,5,6]. Although many elite genes related to multiple agronomic traits such as fiber yield, fiber quality and pathogen resistance were identified, there are still gaps between cotton genomic research in the laboratory and cotton breeding work in the field [7]. Two of these gaps are pedigree formation and the geographical bias of gene resources [6,7,8].

In the present cotton breeding framework, a backbone cultivar is used to hybridize with many other accessions to create elite progenies, forming a pedigree [7,9,10]. A recent study characterized the genetic pattern of genomic segments within an elite cotton pedigree, and featured segments called fingerprint segments of the cotton pedigree were identified by the term frequency–inverse document frequency (TF-IDF) algorithm [7,9,10]. However, this study only characterized the structural variations and ignored the single nucleotide polymorphism (SNP), missing some critical genomic features in this cotton pedigree. Moreover, there is no convenient tool for identifying pedigree fingerprint genomic features.

Apart from the pedigree formation, the geographical bias of gene resources also restricts the utilization of the elite genotypes detected by cotton GWAS research [6,8]. In a previous study, two inversions happened in the upland cotton genome, resulting in the divergence of the modern cotton cohort consisting of about 3000 accessions [6]. In another cotton genomic analysis, researchers found that cultivars with different sources had different contributions to the trajectory of cotton cultivation [8]. In this situation, unique geographical genomic segments contribute to the balance between yield and abiotic stresses [11]. In addition, the genomic variations with region specificity were proved to be involved in environmental adaptation in maize [8]. In this situation, genomic segments with geographical bias contribute to the balance between yield and abiotic stresses. Therefore, detecting genomic features within cultivars from a specific region is crucial for a crop’s genetic analysis. Identifying genomic features from pedigree and cultivars from a particular region is performed by selecting genomic items with a high present frequency in a sub-population (pedigree or cultivars from a specific region) and low present frequency in the background population [7]. The TF-IDF algorithm provides us with a quantitative measurement evaluating genomic items’ conservation in sub-populations and rarity in the background population simultaneously. However, a convenient bioinformatic analysis tool identifying fingerprint genomic items has not been developed.

In this study, we developed FPFinder to identify fingerprint genomic sites in a cotton pedigree and cultivars from various regions [7,12]. We resequenced a cotton pedigree consisting of 20 members by Illumina short reads to construct a cotton pedigree’s SNP map. The background population was a natural cotton cohort with 410 accessions. The 410 accessions were collected from various regions, presenting the adequate genetic diversity of the modern upland cotton cohort cultivated in China. The FPFinder identified 453 pedigree fingerprint sites, and their gene regulatory networks were also constructed to evaluate their roles in cotton development. Based on the information of cultivars from the background population, we identified 12,536 region-specific sites for six regions. The investigation of these region-specific sites revealed their roles in environmental adaptation. The FPFinder enables us to identify fingerprint sites in sub-populations, and we believe that investigations on featured items of sub-populations could provide insights into crop molecular breeding in the future.

## 2. Materials and Methods

### 2.1. Plant Materials

The cotton accessions used in this study are the 20 pedigree members of CRI12, which is an elite upland cotton cultivar reported in the previous study [7]. Among the 20 members, 2 of them are parents of CRI12, Xingtai6871 and Uganda4. The remaining 18 cultivars are progenies of CRI12, including Chuanmian45, Lumianyan16, Yu668, Yumian21, Qinyuan4, Wanmian6, Ekang10, Jinmian33, Sumian9, Xinluzhong7, Handan284, Ekang8, Emian21, Yumian8, Zhongzhi86-6, Yumian11, CRI35 and Jinmian20. The seeds were collected from plants experiencing 4 generations of selfing and cultivated in the greenhouse at room temperature. When three leaves had expanded, they were collected and placed into liquid nitrogen. The collected leaves were stored at −80 °C.

### 2.2. DNA Extraction, Library Construction and Sequencing

The DNA extraction, library construction and DNA sequencing was performed by Novogene (Tianjin, China) according to the pipeline reported previously [12].

### 2.3. Data Filtering, Reads Mapping and Variant Calling

Raw sequence data were stored in the fastq format. Fastp performs a primary filter on the raw data to ensure the quality of the remaining clean data. The clean data of 20 accessions with about a 20–30 × depth was aligned to the CRI12 genome by BWA [13]. After alignment, we used samtools to sort the aligned reads [14]. We used bcftools to perform variant calling and quality control in which variants with a quality score smaller than 30 were trimmed [15].

### 2.4. FPFinder Development

FPFinder consists of 2 parts. The first part of FPFinder is to transform the variant calling file into the variant present|absent table. The second part of the FPFinder calculates the fingerprint score of each site based on the variant present|absent table. The specific use of FPFinder is available at https://github.com/LiuShang-777/FPFinder (accessed on 1 March 2024). For the fingerprint genomic sites in cotton pedigree, we used the permutation method to ensure the threshold of the fingerprint score. In this study, the target population is the CRI12 pedigree containing 20 members, and the background population is the 410 upland accessions reported previously [12]. To set the threshold of the fingerprint score of CRI12’s pedigree, we randomly generated a number ranging from 0 to 20 to simulate the present frequency of a site in the CRI12 pedigree. We also randomly generated a number ranging from 0 to 410 to simulate the present frequency of a site in a background population with 410 upland cotton accessions. The simulated fingerprint score of a site was calculated based on frequencies in the pedigree and 410 accessions. We simulated 1000 times to generate 1000 simulated fingerprint scores and set the top 5% fingerprint score as the threshold. In this study, the threshold of fingerprint sites in cotton pedigree was set as 0.817.

### 2.5. GWAS Analysis

The whole genome sequencing data of about 10 × depth were fetched from NCBI with project number PRJNA399050 [12]. The fiber length of the 410 upland cotton accession was downloaded from http://cotton.hebau.edu.cn/. After read mapping, variant calling and variant filtering described in Section 2.3, we obtained the genomic variation map of 410 upland cotton accessions. Based on the phenotype and genomic variations of 410 accessions, we performed genome-wide association analysis for fiber length. The software EMMAX was applied for GWAS [16]. The threshold of significant variants was set as *p* = 1 × 10^−6^.

### 2.6. Transcriptome Analysis

Transcriptomic analysis in this study is based on CRI12 tissues and multiple tissues of another upland cotton accession, TM-1 [7,17]. Transcriptome data of CRI12 tissues were obtained from Bioproject PRJNA1000640, and transcriptome data of upland cotton’s multiple tissues and abiotic stresses, including cold, hot, drought and salt, were obtained from Bioproject PRJNA490626. The raw data were filtered by Fastp with default parameters [18]. The clean data were aligned to the reference genome of CRI12 by Hisat2 [19]. The alignment files were transformed and sorted by Samtools [14]. Finally, Stringtie was used to quantify gene expression levels [20].

### 2.7. WGCNA Pipeline

The WGCNA package in R was used for weighted gene co-expression network analysis based on the transcriptome of multiple tissues, and a maximum TPM smaller than one among all samples was trimmed [21]. The soft threshold was five because the R square was larger than 0.9. After gene classification, the in-house Python script (available at https://github.com/LiuShang-777/MS_Tool) (accessed on 1 December 2022) was used to link the gene module to the sample type.

### 2.8. Regulatory Network Construction

The regulatory network of a specific gene is constructed based on transcriptome data. The Pearson correlation between the target gene and all genes was calculated by the Pearson function in the Scipy package in Python. The gene pairs with a Pearson correlation larger than 0.7 and a *p*-value smaller than 0.01 were retained as regulatory gene pairs. Cytoscape v3.6 visualized the regulatory network of a single gene [22].

### 2.9. Construction of Phenotype Predicting Model

The transcriptome data of 314 accessions were fetched from NCBI with accessions PRJNA433615 and PRJNA776409 [6,23]. The transcription abundance was estimated by the transcriptome analysis pipeline described above. The expression of the selected genes was used as features, and a support vector machine model was constructed to predict the fiber length based on features of the Scipy package in Python. The Pearson correlation between the predicted value and true phenotype evaluated the performance of the support vector machine.

### 2.10. KEGG Enrichment

The KEGG analysis in this study was performed on CottonFGD (https://cottonfgd.net/, accessed on 1 December 2022) [24].

### 2.11. Statistical Analysis and Data Visualization

The statistical analysis in this study was implemented by the Scipy package in Python. The data visualization in this study was implemented by matplotlib and seaborn packages in Python.

### 2.12. qRT-PCR Experiment

To validate the expression pattern of CRI12_D12G3066, we performed qRT-PCR based on fiber and ovule collected on 15DPA. The fiber and ovule on 15 DPA were collected for cDNA synthesis, and qRT-PCR was performed by TransStart TOP Green qPCR SuperMix (TransGen Biotech, Nanjing, China) on an ABI QuantStudio5 Real-Time PCR System (Applied Biosystems, Foster City, CA, USA). GhHis3 was used as the reference gene for the normalization of relative expression. The forward primer is AGACGAATACGACAC, and the reverse primer is TAAATGGGATCTGTT.

## 3. Results

### 3.1. Developing the FPFinder

**Figure 1:**
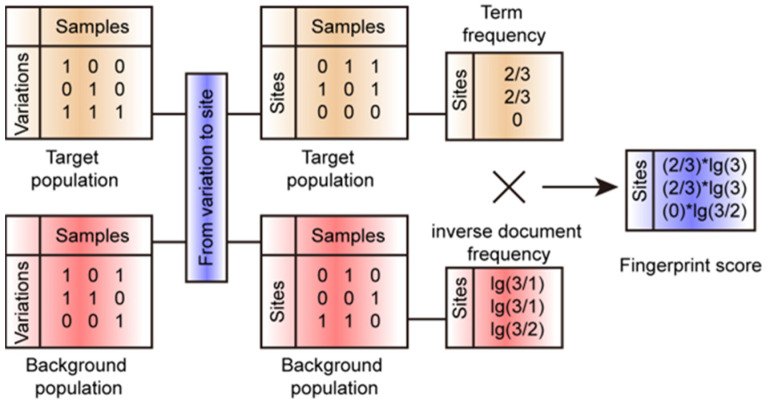
Illustration for workflow of FPFinder. The FPFinder could deal with the files in the format of the variant calling file. It needed the variant distribution information of the target sub-population and the background population to calculate the TF-IDF value as the fingerprint score for each site.

The genetic analysis of population scale is broadly applied to many crops [25,26,27,28,29]. Detecting the fingerprint genomic sites in a specific population is important to understanding the genetic basis of the featured phenotype possessed by a specific population [7,9,10]. Here, we developed an open-source software, FPFinder (Fingerprint Finder), to identify fingerprint genomic sites in a target population based on a comparison of genomic sites’ frequencies between the target population and background population (Figure 1).

The FPFinder consists of the following two steps: input file transformation and fingerprint score calculation. In genetic analysis, the genotype information is usually recorded in the format of a variant calling file (vcf), and the FPFinder transforms these vcf files into the present|absent matrices of genomic sites.

For each genomic site, we calculated the term frequency according to the present frequency in a target population (CRI12 pedigree in this study) and the inverse document frequency in a background population (410 upland cotton accessions in this study) (Figure 1). The fingerprint score of each genomic site was calculated by multiplying the term frequency and inverse document frequency.

### 3.2. Characterization of Fingerprint Genomic Sites in Cotton Pedigree

**Figure 2:**
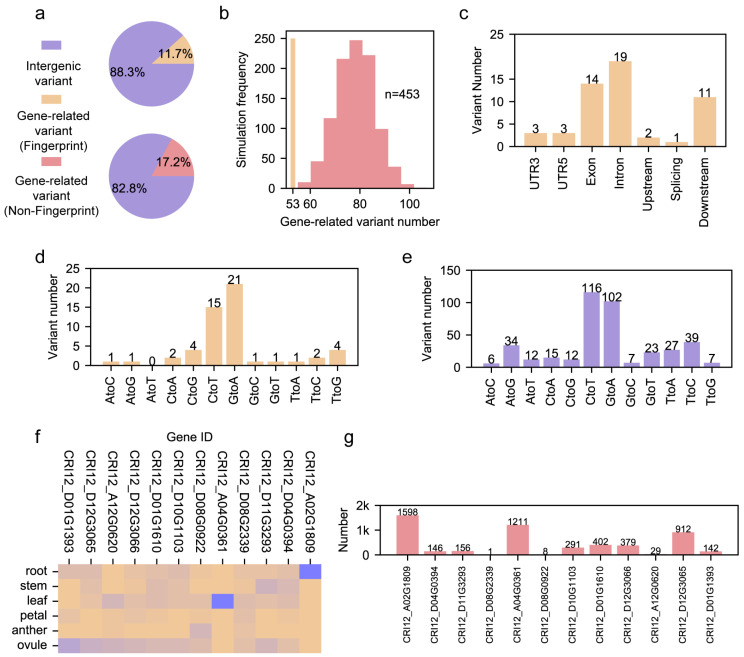
The genomic features of fingerprint genomic sites. (**a**) The ratio of gene-related sites in fingerprint genomic sites and non-fingerprint genomic sites. (**b**) The hist bar for the permutation test for repellence of fingerprint genomic sites. *X*-axis represents the number of gene-related sites in the down-sampled non-fingerprint sites. The *Y*-axis represents the frequency of events in 1000 permutations. (**c**) The genomic annotation of gene-related pedigree fingerprint genomic sites. (**d**) The number of 12 SNP types in gene-related sites from pedigree fingerprint genomic sites. (**e**) The number of 12 SNP types in intergenic sites from pedigree fingerprint genomic sites. (**f**) Heatmap of 12 genes among 6 cotton tissues; the blue color represents the high expression level, while the brown color represents a low expression level. (**g**) The number of genes from 12 gene regulatory networks.

Pedigree is an important form of accession evaluation in modern cotton breeding [7,9,10]. A cultivar with elite agronomic traits is selected as the backbone cultivar for the creation of new accessions. Although the fingerprint segments within the pedigree of an elite cultivar, CRI12, have been identified, the pedigree’s fingerprint genomic sites, which presented as single nucleotide polymorphisms (SNPs), were ignored in the previous study [7]. We resequenced the previously reported pedigree with 20~30× Illumina short reads to construct the high-quality SNP map. To identify the fingerprint genomic sites in CRI12’s pedigree, FPFinder demands the construction of the background population’s SNP map. In this study, a previously reported population consisting of 410 accessions collected from various regions was used as the background population [12]. We identified 453 fingerprint genomic sites from 6,320,340 SNPs within the CRI12 pedigree (Table S1). Based on the genomic annotation by Annovar, we found that 11.7% (53 genomic sites) of the fingerprint genomic sites were gene-related, located in UTR3, UTR5, exon, intron, splicing site, upstream 1 kb and downstream 1 kb of a gene (Figure 2a). By contrast, the non-fingerprint genomic sites had a higher ratio of gene-related type, taking up 17.2% (Figure 2a). Because the number of fingerprint genomic sites is limited, we randomly selected 1000 sets of non-fingerprint genomic sites, and each set contained 453 sites. We calculated the number of gene-related genomic sites for each set. From the comparison between gene-related sites with fingerprint and non-fingerprint categories, we found that all one thousand batches of the non-fingerprint category had more gene-related genomic sites than those in the fingerprint category (Figure 2b). This result indicates that conserved genomic sites in the cotton pedigree tend to be in an intergenic region and away from coding and regulatory regions. Given this, we inferred that pedigree unique genes may have suffered extreme selection because the variants within them are rare in the modern cotton cohort with 410 accessions.

We checked the genomic annotation of 53 pedigree fingerprint sites. We found that more than 80% (44 of 53) of sites were located in the exon, intron and downstream 1 kb from a gene. There were only 3, 3 and 1 sites in the UTR5, UTR3 and splicing sites, respectively (Figure 2c). In addition, we characterized the variation feature of pedigree genomic sites within the gene region and intergenic region (Figure 2d,e). We found that G->A and C->T types were the most abundant in the sites of both gene and intergenic regions. However, we found that the number of G->A types was larger than that of C->T in sites within the gene region, while the pattern contrasted in sites within the intergenic region. Apart from the above two variation types, we also found the divergence of other variation types between genomic sites within the gene region and intergenic region. For instance, T->G had a higher ratio in pedigree genomic sites within the gene region than in pedigree genomic sites within the intergenic region. This divergence indicates that the mechanism of variant formation may differ between pedigree genomic sites within the gene and intergenic regions.

We checked the distribution of 53 gene-related pedigree fingerprint sites and found that 12 genes in the CRI12 pedigree were influenced by this. We further characterized the transcriptomic landscape of these twelve genes in six tissues which are essential for plant development, including root, stem, leaf, petal, anther, and ovule (Figure 2f). We found that these genes had diverse transcription patterns, and several of them had important biological functions (Table S2). *CRI12_D01G0610* encodes protein NAP1, which is involved in the regulation of actin and microtubule organization [30]. *CRI12_A04G0361* encodes the XA21 protein, which is conferred as a crucial factor in plant innate immunity in rice [31]. *CRI12_D11G3293* encodes the protein SEC24A, which regulates cell size and male gametophyte development [32,33]. *CRI12_D12G3065* encodes protein GB1, playing a role in hormone-mediated cell division [34]. The gene annotation showed that these genes influenced by CRI12 pedigree fingerprint sites had diverse effects and were involved in various biological processes. We further constructed the gene regulatory networks (GRNs) based on the transcriptome data of six tissues (Table S3). We noticed that the number of genes in 12 GRNs varied from 1 to 1598 (Figure 2g). We noticed that *CRI12_D12G3066* and *CRI12_D08G2339* encode the MYB transcription factor and RNA helicase, respectively. These two proteins have a role in DNA binding and RNA pre-processing, showing their potential role in the formation of GRN, while GRNs of the other genes encoding enzymes may be formed by incorporation with genes belonging to the same biological pathways (Table S4). Although proteins encoded by *CRI12_D08G0922* and *CRI12_A12G0620* have not been annotated, they still have their own GRNs (Table S3). Different from other GRNs, the genes in these two GRNs have no biological pathway enriched, indicating that the roles of *CRI12_D08G0922* and *CRI12_A12G0620* need to be further investigated in the future.

### 3.3. Role of Pedigree Fingerprint Site-Related Genes in Cotton Development

**Figure 3:**
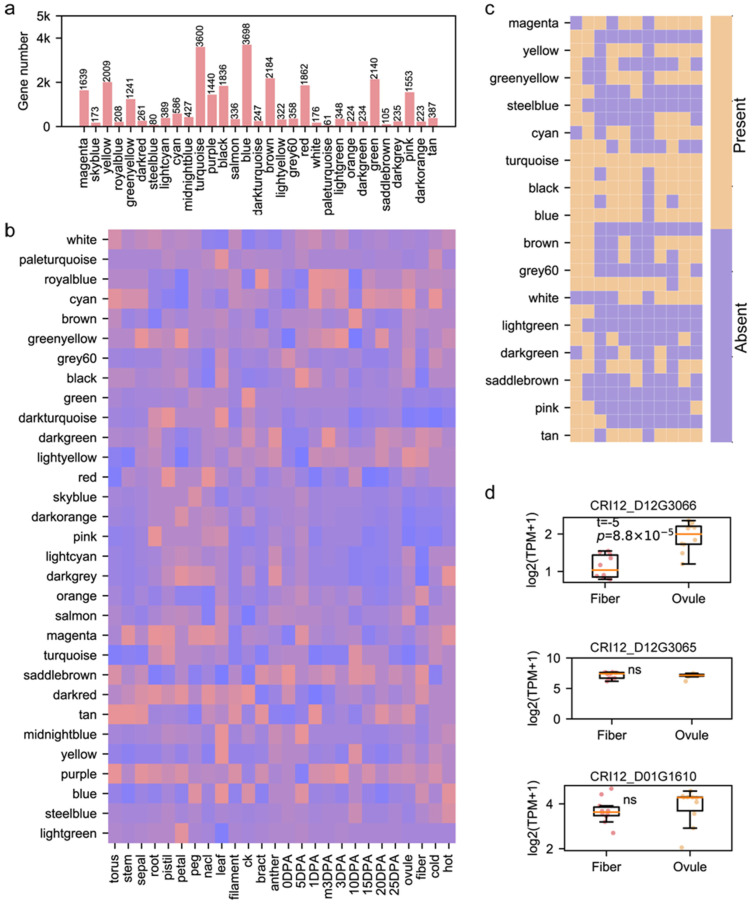
The gene regulatory networks of genes influenced by pedigree fingerprint sites. (**a**) The number of genes in each gene module generated from WGCNA. (**b**) The linkage between gene modules and the phenotypes; the red color represents high correlation, while the blue color represents low correlation. (**c**) The present|absent heatmap characterizing the distribution of GRNs influenced by pedigree fingerprint sites. (**d**) The boxplot for expression levels of three genes between ovule samples and fiber samples from -3DPA-25DPA. The *t*-test was used.

Since we obtained 12 fingerprint site-related genes in the CRI12 pedigree and their corresponding GRNs, we could explore the role of 12 genes and GRNs in cotton development. We constructed a weighted gene co-expression network based on 137 samples of upland cotton (including multiple tissues and samples under abiotic stresses) according to the WGCNA pipeline part in the Methods section (Table S5). Finally, we identified 32 gene modules from co-expression construction and found that the gene number from different modules varied greatly, from 61 to 3698 (Figure 3a, Table S6). We performed the association analysis to link modules and phenotypes based on the Pearson correlation. We noticed that modules had diverging module–phenotype linkage patterns (Figure 3b). For instance, the light-green module had a tight linkage with petal tissue; the yellow module was highly associated with the leaf and fiber on 10 DPA (day post anthesis); and purple and dark-red modules were related to multiple tissues. These results indicated that various gene modules had different functions, providing a platform for the investigation of pedigree fingerprint site-related GRNs’ role in cotton development. We checked the presence/absence of genes from pedigree fingerprint site-related GRNs (Figure 3c). The genes of *CRI12_D08G0922*’s GRN only presented in red and turquoise modules, while the genes of *CRI12_A02G1809*’s GRN presented in almost all gene modules except for magenta, cyan, white and dark-green modules. *CRI12_A02G1809* encodes cytochrome P450, an important protein involved in many biological processes, and this may result in the broad distribution of *CRI12_A02G1809*’s GRN among gene modules [35,36,37]. Cotton fiber is the most important agronomic trait, and we inferred that some of the 12-pedigree fingerprint site-related genes could influence cotton fiber development. Considering that fiber originates from a single cell on the ovule epidermis, we compared the gene expression of 12 genes between the ovule and fiber during fiber development. Three of them had expression during fiber development (the maximum expression value among fiber and ovule samples is larger than 1), and *CRI12_D12G3066*, a gene encoding the MYB transcription factor, had significantly higher transcription abundance in the ovule compared to fiber (*t*-test, *p* = 8.8 × 10^−5^) (Figure 3d). The transcription pattern of *CRI12_D12G3066* was also validated by qRT-PCR in fiber and ovule on 15 DPA (Figure S1). The role of the MYB family in fiber development has been thoroughly illustrated in a set of previous studies [38,39,40,41]. The above results show that pedigree fingerprint site-related genes are involved in many complex biological processes and could be a precious gene resource for molecular breeding.

### 3.4. Geographical Bias of Functional Sites Results in Fiber Length Divergence

**Figure 4:**
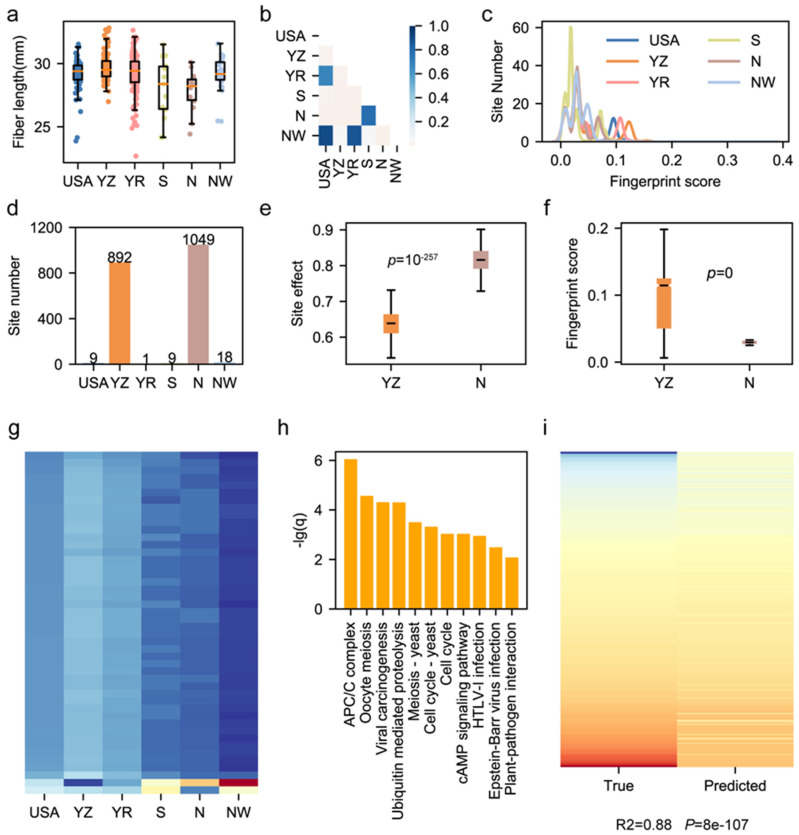
Characterization of geographical bias of a fiber-length-related sites. (**a**) Boxplot for fiber length of cultivars from 6 regions. (**b**) The *p*-values of the *t*-test on comparisons between 2 different regions. The shallow yellow color represents *p*-values smaller than 0.05 in the *t*-test. (**c**) Distribution of fingerprint scores possessed by fiber-length-related genomic sites in 6 regions. (**d**) The number of fiber-length-related genomics sites enriched in each region. (**e**) Phenotypic effects of fiber-length-related genomic sites enriched in the Yangze River region and North China. A *t*-test was used. (**f**) The fingerprint scores of fiber-length-related genomic sites enriched in the Yangze River and North China. A *t*-test was used. (**g**) Heatmap of fingerprint scores of 46 fiber-length-related sites of region-specificity. The blue color means a low fingerprint score, while the red color means a high fingerprint score. (**h**) The KEGG enrichment results of 12 genes influenced by 46 fiber-length-related sites. (**i**) The heatmap for true phenotype value and the predicted value generated by the expression-supported prediction model. Pearson correlation was calculated, and the chi-square test was performed.

The modern upland cotton cohort in China consists of accessions of high genetic, phenotypic and geographical diversities [38,39,40,41]. Previous genetic research revealed that large inversions caused the geographical divergence of cultivated cotton accessions [5,6,8,42,43]. We inferred that functional genomic sites may also have geographical bias and result in phenotypic divergence among cultivars from different regions. In this study, we utilized a reported natural population consisting of 410 upland cotton accessions and aligned their whole genome sequencing data to the CRI12 genome to construct the modern cotton cohort’s SNP variome. The cultivars in this cohort are mainly from the following six regions: the USA, the Yangtze River region in China (YZ), the Yellow River region in China (YR), the South region in China (S), the North region in China (N), and the Northwestern region in China (NW) (Table S8). We characterized the fiber length (FL) among 410 upland cotton cultivars. The fiber lengths of cultivars from six regions showed divergence (Figure 4a). The *t*-test results of the fiber length comparison among six regions showed that cultivars from the YZ region had a longer fiber length than those from other regions (Figure 4b). To ensure the relationship between the geographical bias of functional sites and phenotypic divergence of cultivars from various regions, we performed a genome-wide association study (GWAS) according to the Methods section and detected 2646 functional sites for FL. Based on the cotton cohort variome, we calculated the functional sites’ fingerprint score using FPFinder. We found a correlation between the site’s geographical bias and the region-specific divergence of the phenotype. For instance, we noticed that cultivars from the YZ sub-population had a higher fiber length than that of cultivars from the other sub-populations (Figure 4a,b), while functional sites had a higher fingerprint score in YZ sub-populations (Figure 4c). Among the 2646 functional sites related to fiber length, we extracted genomic sites that had positive effects on fiber length and found that 892 of them had the highest fingerprint score in the YZ sub-population, while 1049 of them had the highest fingerprint score in the N sub-population (Figure 4d). We compared the phenotypic effects of sites which had the highest fingerprint score in YZ and N sub-populations and found that those which had the highest fingerprint score in the N sub-population had larger phenotypic effects (*t*-test, *p* = 1 × 10^−257^) (Figure 4e). However, the comparison between the fingerprint scores of functional sites belonging to two sub-populations showed that higher fingerprint scores were possessed by functional sites enriched in the YZ sub-population (*t*-test, *p* = 0) (Figure 4f). This result indicates that the enrichment of elite YZ-specific sites improves the fiber length of cultivars from the Yangtze River region.

We further annotated 196 fiber-length-related sites within the gene region (Table S10). After trimming genomic sites whose maximum fingerprint score across six sub-populations was smaller than 0.1, we obtained 46 region-specific fiber-length-related sites, and most of them were enriched in YZ and YR sub-populations (Figure 4g). These 46 region-specific genomic sites influenced 12 genes which were enriched in 11 biological pathways, including the APC/C complex, signaling pathway and pathogen-related terms, implying the complex mechanisms of these genes for improving the fiber length (Figure 4h). Finally, to validate the utility of the 12 genes in cotton breeding, we utilized the transcriptome data of 314 accessions from two independent works of research to construct a phenotype prediction model [6,23]. The model was built by the support vector regression method in which the expression levels of 12 genes in fiber at 15 DPA were used as features, and the fiber length was used as the label. Finally, the fiber length prediction model had an elite performance (R^2^ = 0.88, *p* = 8 × 10^−7^), indicating that these 12 genes could be used for fiber improvement in the future (Figure 4i).

### 3.5. Role of Region-Specific Genomic Sites in Cotton Development

**Figure 5:**
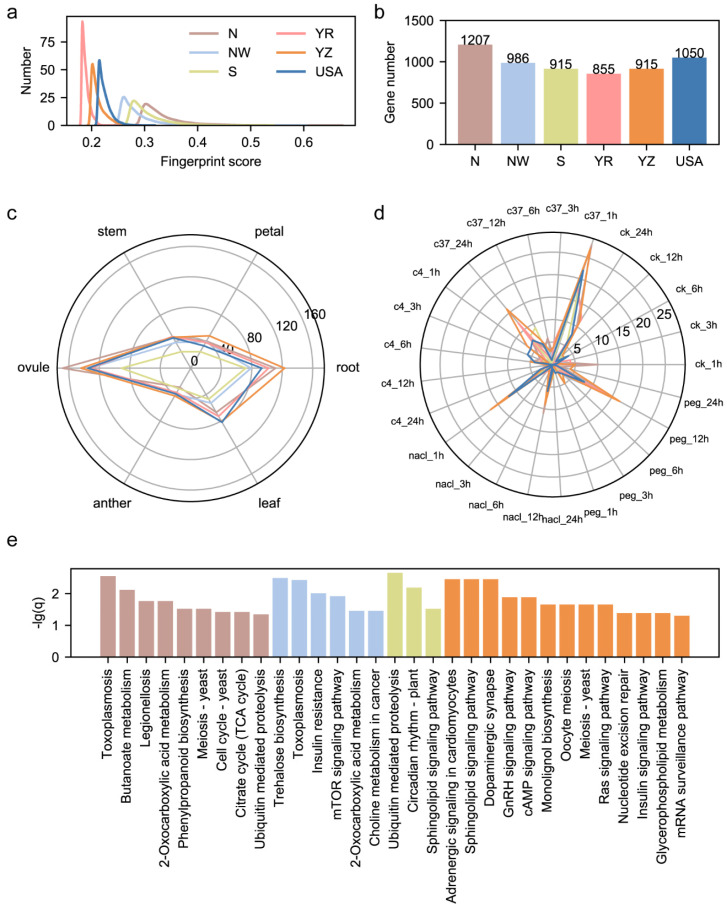
Characterization on genomic sites of high-region specificity. (**a**) Distribution of fingerprint scores of identified region-specific genomic sites in 6 regions. (**b**) The number of genes influenced by region-specific genomic sites in 6 regions. (**c**) The radar plot on predominantly expressed genes among 6 tissues. (**d**) The radar plot on predominantly expressed genes among 4 abiotic stresses. (**e**) KEGG enrichment results of genes influenced by region-specific genomic sites.

Through the calculation of fingerprint scores for functional sites, we revealed that the geographical bias of functional sites results in the region-specific divergence of the fiber length. However, we noticed that the fingerprint scores of the fiber-length-related genomic sites are always smaller than 0.2 (Figure 3c). Here, we scanned the genomic sites all over the modern cotton cohort and calculated the fingerprint score for each sub-population. We selected sites with the top 0.1% fingerprint scores as the region-specific genomic sites, and 12,536 fingerprint genomic sites were filtered out for each sub-population. We found that among 12,536 region-specific sites, most of them had fingerprint scores larger than 0.2, exhibiting higher region specificity than fiber-length-related genomic sites (Figure 5a). We annotated these genomic sites of high-region-specificity and detected genes specifically influenced by them. We found that fingerprint sites from N, NW, S, YR, YZ and USA sub-populations were located in 1207, 986, 915, 855, 915 and 1050 genes and their promoters (Figure 5b). We explored these genes’ transcriptional patterns among six tissues and found that most genes were predominantly expressed in the ovule and root, indicating that the root and ovule may be two important tissues in crop adaptation to various environments (Figure 5c). We further checked the expression pattern of these genes on abiotic stress-related transcriptome and found that, except for cold stress, these region-specific genes were predominantly expressed under drought stress, hot stress and salt stress, showing the ability of region-specific genes to environmentally adapt (Figure 5d). Performing KEGG enrichment on region-specific genes for each sub-population, we noticed that except for the YR and USA sub-population, genes from the remaining four subpopulations had crucial biological pathways enrichment (Figure 5e). For example, ubiquitin-mediated proteolysis was found in the enrichment results of genes from both N and S sub-populations, implying that genes from this pathway may play an important role in resistance to abiotic stresses and environmental adaptation.

## 4. Discussion

In this study, we developed a fingerprint site identification software, FPFinder, for the crop genomics community. Based on the FPFinder, we detected fingerprint genomic sites within an elite cotton pedigree based on the Illumina short reads. Further investigations on pedigree fingerprint genomic sites characterized the genomic features of pedigree fingerprint sites and revealed the role of these pedigree fingerprint sites in cotton development. Apart from the cotton pedigree, we also utilized FPFinder to evaluate the geographical bias of fiber-length-related genomic sites in a natural population and identified region-specific genomic sites. We found a relationship between unique geographical functional sites and phenotype divergence among cotton cultivars from various regions. Through the identification of region-specific sites, we also found that genomic sites enriched in a specific region might play a role in environmental adaptation.

Pedigree formation is a common phenomenon in crop breeding [7,9,10,44,45,46,47,48,49]. Our previous study detected the featured structural variations within an elite cotton pedigree, the pedigree of CRI12, based on long-reads sequencing [7], while the fingerprint genomic sites within this cotton pedigree could not be characterized by long reads due to their low single base sequencing accuracy. The pedigree fingerprint sites identified by FPFinder show similar genomic annotation with the fingerprint segments identified previously, with fewer in gene-related regions, indicating that these highly conserved genomic items in cotton pedigrees repel the gene-related region, and the genes influenced by these genomic items might have undergone intense selection [7]. The geographical bias of genotype distribution has been proven as the essential factor for phenotypic divergence [6,8,11]. In cotton, two large genomic inversions were regarded as the causal factor of upland cotton’s geographical divergence [6]. In a natural population, we identified the genomic sites with geographical bias using the FPFinder and revealed that several elite sites had a higher fingerprint score in cultivars from the Yangze River region, the fiber length of which is longer than cultivars from other regions. Investigations on geographical bias among the cotton natural population indicated that although genomic resources of high-region specificity did not contribute to fiber length directly, they played roles in cotton’s environmental adaptation.

The threshold of the fingerprint score is flexible. This was a conservative way to ensure the threshold was a permutation-based method applied in pedigree analysis in this study [50,51,52,53,54]. However, the threshold calculated by the permutation-based method was strict in some scenes such as the sub-population analysis based on GWAS in this study. The user could set the threshold of the fingerprint score according to their own results, and the false positive results could be controlled by adding more information, such as transcriptomic data. In my own opinion, the type I error (false negative result) should be avoided, while the type II error (false positive result) is inevitable. Additional information from other dimensions, such as transcriptomic data and metabolomic data, could be utilized to further filter the results and correct the type II error with a loose threshold, while some crucial results will be missed under a strict threshold in analysis. Thus, the threshold of fingerprint items depends on the FPFinder’s results and the user’s demands.

As a large amount of genomic data is released, genomic analysis on a population scale is a trend in cotton genomics [55]. To identify functional genes, GWAS could not fulfill the analysis demands of future cotton genomics, and the characterization of features of a specific population, such as pedigree or cultivars from a specific region, could help us to figure out more genomic items with biological meanings. Genomic features of a sub-population are always conserved within a subset population but undergo a sweep selection in large background populations. Recent genomic research across species showed that conserved genomic variations possess huge phenotypic effects due to their tight evolutional constraints [55]. Compared to the significant sites with phenotypic effects in GWAS, fingerprint sites are rare in the large cohort, which is used as the background. Therefore, the fingerprint sites are trimmed in GWAS because of their extremely minor allele frequencies. In genomic analysis, fingerprint sites are complementary to significant sites in GWAS. We believe that the combination of fingerprint sites and significant sites in GWAS characterize a more detailed landscape of functional genomic variations.

## 5. Conclusions

We developed the FPFinder to identify fingerprint genomic items within subset cotton cohorts. The detection of fingerprint genomic items could benefit from genetic analysis and molecular breeding broadly in other crops.

## Data Availability

The Illumina resequencing data of 20 pedigree members will be available according to the request to the corresponding author Guoli Song.

## Supplementary Materials

The following supporting information can be downloaded at: https://www.mdpi.com/article/10.3390/genes15030378/s1, Figure S1: The qRT-PCR validation for CRI12_D12G3066 between ovules and fibers. Table S1. The genomic annotation of 453 fingerprint genomic sites in cotton pedigree. Table S2. The twelve genes influenced by pedigree fingerprint genomic sites. Table S3. The gene regulatory network of 12 genes based on Pearson correlation. Table S4. The KEGG enrichment of genes in 12 gene regulatory networks. Table S5. The information about sample and transcriptomic data of WGCNA. Table S6. The module classification of WGCNA. Table S7. The number of genes in 12 genes’ GRNs among different modules. Table S8. Geographical information about 364 upland cotton accessions. Table S9. The *p*-value, effects, and fingerprint score of each fiber-length-related genomic site. Table S10. The fiber-length-related genomic sites within coding genes. Table S11. The selected 12 genes for constructing the expression-supported phenotype prediction model.
