# Supplementary figures and images for: Fingerprint Finder: Identifying Genomic Fingerprint Sites in Cotton Cohorts for Genetic Analysis and Breeding Advancement
Source: Genes (Basel). 2024 Mar 19;15(3):378. doi: 10.3390/genes15030378 (PMC10970022; doi:10.3390/genes15030378)

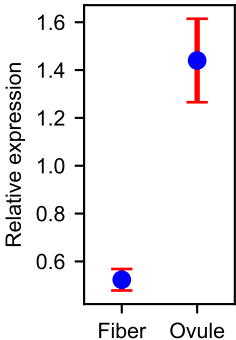

Supplement: Supplementary file 1 [file genes-15-00378-s001.zip › Figure S1.pdf]
